# Food Allocation under Asynchronous Hatching Conditions of Great Tits (*Parus major*)

**DOI:** 10.3390/ani13091443

**Published:** 2023-04-23

**Authors:** Ji-Won Kang, Jong-Koo Lee

**Affiliations:** Division of Life Sciences, College of Life Sciences and Bioengineering, Incheon National University, 119 Academy-ro, Yeonsu-gu, Incheon 22012, Republic of Korea; jiwonkang@inu.ac.kr

**Keywords:** hatching asynchrony, brood reduction, breeding strategies, food allocation rules, nestling provisioning

## Abstract

**Simple Summary:**

In some species of birds, asynchronous hatching occurs when incubation begins before completion of clutch, resulting in age differences between nestlings. According to the brood reduction hypothesis, which explains the reason for asynchronous hatching, selective survival is permitted only for older nestlings when availability of food is unpredictable. Due to the feature of food distribution, passerines are not likely to practice selective feeding of older nestlings. The essential purpose of this study is to determine whether the brood reduction hypothesis can explain asynchronous hatching in passerines. Infrared cameras were installed inside nest boxes to determine whether great tit (*Parus major*) parents, which show asynchronous hatching, practices selective feeding of older nestlings. The results of this study showed that great tit parents did not practice selective feeding of older nestlings, highlighting that using the brood reduction hypothesis to explain asynchronous hatching of great tits is not reasonable. Further research is needed in order to explain the adaptiveness of asynchronous hatching.

**Abstract:**

The brood reduction hypothesis, which explains asynchronous hatching in birds, as an adaptation that enables selective survival of older nestlings when availability of food is unpredictable. This study was conducted in order to determine whether the brood reduction hypothesis can explain asynchronous hatching in passerines. Infrared cameras were installed inside nest boxes where great tits (*Parus major*) were attempting to reproduce in order to determine whether the parents practiced selective feeding of older nestlings. According to the results of the study, no significant difference was observed between the hatching order and the average number of feedings per nestling. In addition, when examining the distribution of food according to hatching order over time, every 30 min, beginning at 9 a.m., selective distribution of food to older nestlings was not observed. In conclusion, use of the brood reduction hypothesis, which supports selective provision of beneficial feeding of older and larger nestlings, to explain the asynchronous hatching of passerines is problematic, thus conduct of future studies focusing on other hypotheses in order to explain the asynchronous hatching of this passerine bird will be necessary.

## 1. Introduction

In some species of birds asynchronous hatching occurs when incubation begins before completion of clutch, resulting in age differences between nestlings [1,2,3,4,5,6,7]. Many hypotheses have been proposed to explain asynchronous hatching. According to the brood reduction hypothesis, only older nestlings are targeted for selective survival when the availability of food is unpredictable [1,2]. The brood reduction hypothesis explains asynchronous hatching as an adaptation that increases fledging success when predicting the amount of food that can be fed to nestlings is difficult. In an environment of food scarcity, older nestlings are selectively permitted to survive, so that clutch size is reduced and at least some nestlings survive [8,9,10,11,12].

Several studies have attempted to determine whether the brood reduction hypothesis can explain asynchronous hatching in various species. For birds of prey, hunting larger prey is opportunistic. In addition, because of the type of food, the number of prey deliveries is small, and there is fierce competition among siblings for food provided by parents [13,14]. Therefore, an adequate amount of prey may not be available for birds of prey in an environment of food scarcity. Thus, the asynchronous hatching of birds of prey can be explained using the brood reduction hypothesis [15]. In studies of black-billed magpies (*Pica pica*) [16] and house wrens [17], asynchronous hatching is explained using the brood reduction hypothesis, as well for passerines. However, because passerines consume small-sized prey, nestlings are able to swallow their prey at once; thus, there is less competition between siblings compared to birds of prey [18,19,20,21]. For species whose asynchronous hatching can be explained using the brood reduction hypothesis, selective feeding of older nestlings is essential in order to increase the rate of successful breeding through asynchronous hatchings. However, due to the feature of food distribution, passerines are not likely to practice selective feeding of older nestlings; thus, it is necessary to determine whether the brood reduction hypothesis can explain asynchronous hatching of passerines [15].

The purpose of this study is to analyze the variables associated with the food distribution practices in a passerine and to determine whether passerines practice selective feeding of older nestlings. Several studies of birds of prey [22,23,24] have reported on feeding of older nestlings; however, studies on passerines have rarely been reported [15]. Our hypothesis that passerines do not give priority to feeding their older nestlings was tested in this study by the analysis of the food distribution behavior of great tits (*Parus major*). The average clutch size of great tits ranges from three to eleven and is, therefore, suitable for the observation of food distribution behavior resulting from asynchronous hatching [25,26,27] that occurs in this species [28,29]. An analysis of the food distribution behavior of great tits was performed, and an analysis of hatching order in relation to food distribution, beak-open order, nestling age, nestling location, and date factors was also performed for examination of the prey distribution practices of great tits.

## 2. Materials and Methods

### 2.1. Fieldwork

This study was conducted at Incheon National University (35°22′15.042′~35°22′45.264′ N, 126°37′37.542′~126°38′15.3456′ E) in the western part of Korea from March to July, the breeding season for great tits, in 2020, 2021, and 2022. Great tits, which are native to Asia and Europe [30], are secondary cavity-nesting birds [31,32] suitable for research in nest boxes. Twenty-nine nest boxes were installed in February 2020; nest boxes were monitored for confirmation of breeding attempts from the end of March, when egg laying begins. Discovery of more than one egg was defined as a breeding attempt [33]. For recording inside the dark nest box, a camera (Wildlife Wi-Fi Bird Box Camera 3rd Gen, Green Feathers, Bristol, United Kingdom) with a capacity for infrared recording was installed in the nest where attempted breeding had been confirmed. To determine the hatching order of nestlings, we marked with colored paint each nestling’s head and back according to hatching order, and the number of nestlings was determined according to the date of hatching and the hatching order.

### 2.2. Statistical Analysis

**Figure 1:**
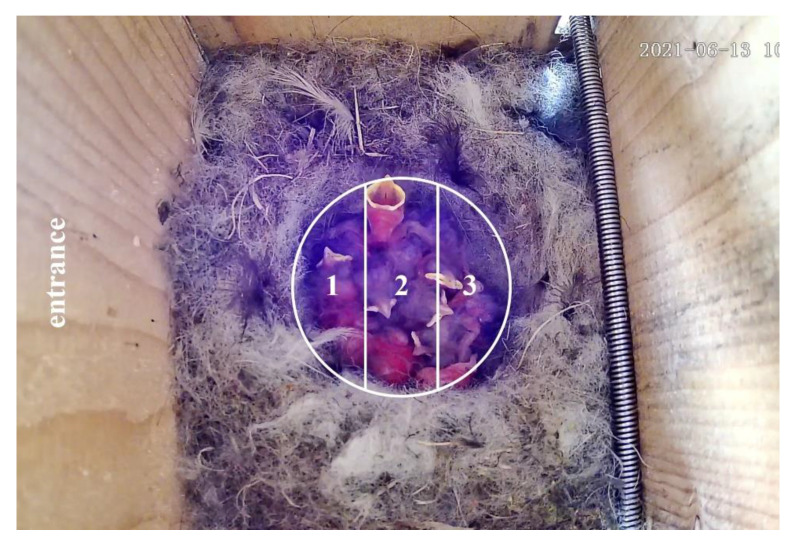
A photograph divided into three sections based on the distance between the entrance to the nest box through which the parents enter and the location of a nestling that has been fed. In the photograph, the entrance to the nest box is located on the left side.

Analysis of the data was performed separately for nests that showed asynchronous hatching over two days (*n* = 8) and nests that showed asynchronous hatching over three days (*n* = 10). Day 0 was regarded as the day when all nestlings hatched; an analysis of the main food distribution time from day 1 to day 6, with recordings from 9:00 am to 12:00 am, was performed using a viewer (GOMPlayer, media player for Windows, GOM & Company, Seoul, Republic of Korea) which enables frame-by-frame inspection. The following factors were analyzed: (1) Hatching order according to asynchronous hatching; nestlings hatched on the same day received the same marking. Nestlings born on the first day were marked as 1, nestlings born on the second day were marked as 2, and nestlings born on the third day were marked as 3. (2) The age of each nestling by day, with day 0 designated as the day each nestling hatched. (3) Beak-open order, coded as 1 if the nestling who ate was the first among the siblings to open its beak when eating, or 0 otherwise. (4) Position of the nestling in relation to the entrance of the nest box (Figure 1) was coded as 1 when the nestling was fed close to the nest’s entrance, and as 3 when it was fed furthest from the entrance. (5) The date for determining the relationship between the start of breeding season and food supply. When the camera was covered by the bodies of the parents (15% of feeding occurrences), data that could not be used in determining which nestling was fed were excluded from statistical analysis.

Multiple linear regression analysis was performed in order to examine the association between the five factors, hatching order, beak-open order, nestling age, nestling location, date, and the average number of feedings per nestling. An independent sample test was performed for the average number of feedings from day 1 to day 6, and a comparison between nests that hatched over two days and nests that hatched over three days. An independent sample test was performed to determine the average number of feedings per nestling depending on whether the beak was opened first. An independent sample t-test was performed for nests that hatched over two days, and a one-way ANOVA was performed for nests that hatched over three days, followed by a post hoc test (Scheffe) in order to determine whether there was a significant difference between the average rate of feeding per nestling, according to the hatching order and the date after all nestlings had hatched. An independent sample t-test was performed for nests that hatched over two days, and a one-way ANOVA was performed for nests that hatched over three days, followed by a post hoc test (Scheffe) in order to determine whether there was a significant difference between the average number of feedings per nestling, according to hatching order and time, every 30 min, beginning at 9 a.m. Statistics were analyzed using IBM SPSS Statics 25.

## 3. Results

In nests that hatched over two days, the average for the first hatched nestling was 4.75 ± 1.98 and the average for the second hatched nestling was 3.13 ± 1.97, with a clutch size of 7.88 ± 0.60; in nests that hatched over three days, the average for the first hatched nestling was 3.80 ± 1.83, the average for the second hatched nestling was 3.00 ± 1.67, and the average for the third hatched nestling was 1.20 ± 0.40, with a clutch size of 8.00 ± 1.26 (Table 1). The average number of feedings per nestlings showed that the average was 20.54 ± 11.56 for nests that hatched over two days and 23.05 ± 9.39 for nests that hatched over three days (Table 1). The results of determining the average number of feedings from day 1 to day 6 showed that on the day after hatching of all nestlings (from 09:00 a.m. to 12:00 a.m., during the day), the average was 123.25 ± 47.07 for nests that hatched over two days and 138.30 ± 30.11 for nests that hatched over three days (an independent sample test, t = −0.775, *p* = 0.449) (Table 1).

**Figure 2:**
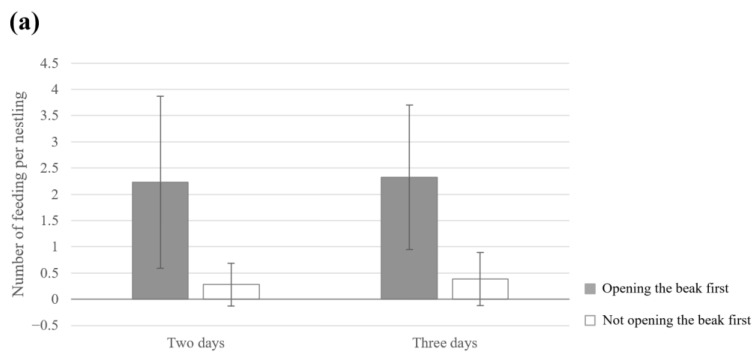

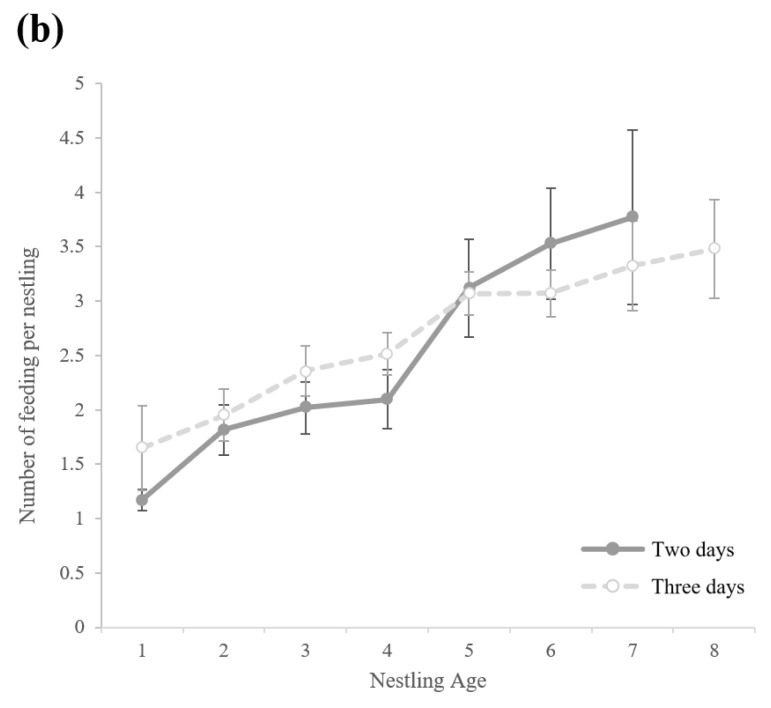
(**a**) The average number of feedings per nestling during the period from 9:00 a.m. to 12:00 a.m. depending on whether the chick being fed is the first one of the brood to open its beak when the adult arrived, in nests that hatched over two days and nests that hatched over three days. Each value was rounded to the fifth decimal place (an independent sample test, nests that hatched over two days: t = 11.308, *p* < 0.001, nests that hatched over three days: t = 17.696, *p* < 0.001). (**b**) In nests that hatched over two days and nests that hatched over three days, the average number of feedings per nestling during the hours from 9:00 a.m. to 12:00 a.m. according to nestling age (days). The solid lines indicate nests that hatched over two days, and the dotted lines indicate nests that hatched over three days. For all nestlings, each hatch day is day 0, data from 1 to 8 days (data from nests that hatched over two days are from 1 to 7 days). Bars indicate the standard error.

The results of multiple linear regression analysis demonstrated that hatching order was not a significant factor in regard to the average number of feedings per nestling for both nests that hatched over two days and nests that hatched over three days (Table 2 and Table 3). Beak-open order and nestling age were significant factors in regard to the average number of feedings per nestling (Table 2 and Table 3). A significantly higher average number of feedings per nestling was observed for nestlings that opened their beaks first compared with those that did not (Figure 2a). In addition, the average number of feedings per nestling increased with the increasing age of each nestling (Figure 2b). The other two factors (nestling location and date) were not significant in regard to the average number of feedings per nestling.

**Figure 3:**
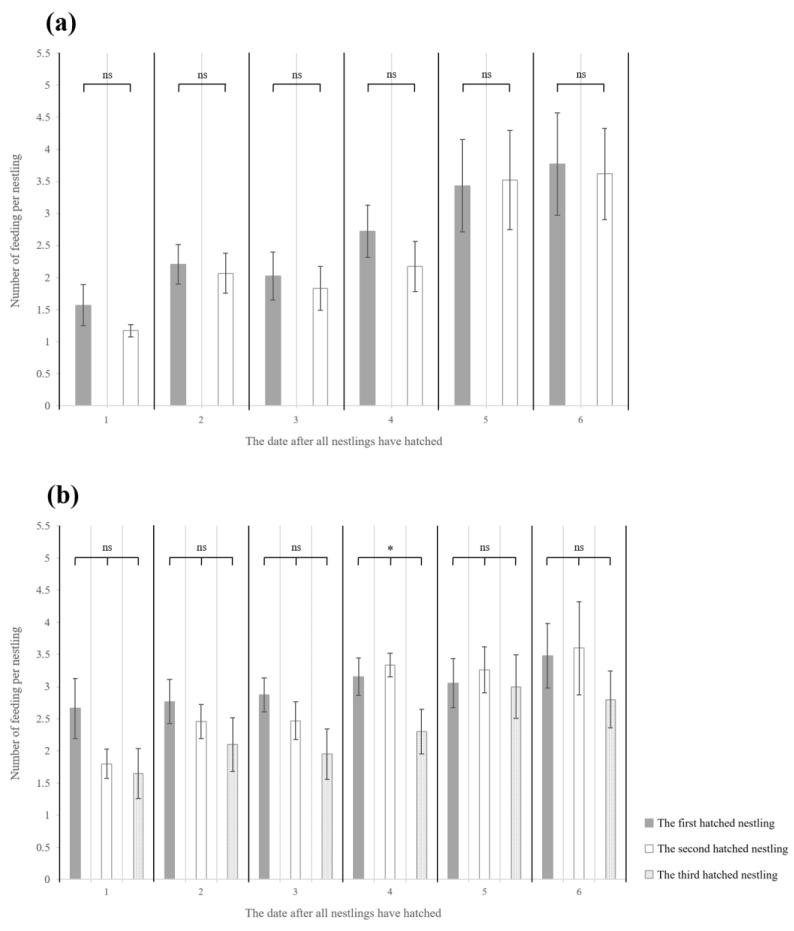
The average number of feedings per nestling during the period from 9:00 a.m. to 12:00 a.m., according to hatching order and date (days) after all nestlings had hatched. (**a**) Nests that hatched over two days, (**b**) nests that hatched over three days. From day 1 to day 6, all nestlings hatched on day 0. The black bar represents the first hatched nestling, the white bar represents the second hatched nestling, and the gray bar represents the third hatched nestling. (ns > 0.05, * *p* < 0.05).

Despite an increase in the average number of feedings per nestling with the increasing age of nestlings in both nests that hatched over two days and nests that hatched over three days, no change in the average number of feedings per nestling was observed according to hatching order (Figure 3a,b). There was no statistically significant difference for each day in nests that hatched over two days (Figure 3a). In nests that hatched over three days, a significant difference (*p* = 0.047) between hatching order was observed on day 4. However, the results of the post hoc test (Scheffe) showed no significant difference; thus, no substantial difference was demonstrated (Figure 3b).

**Figure 4:**
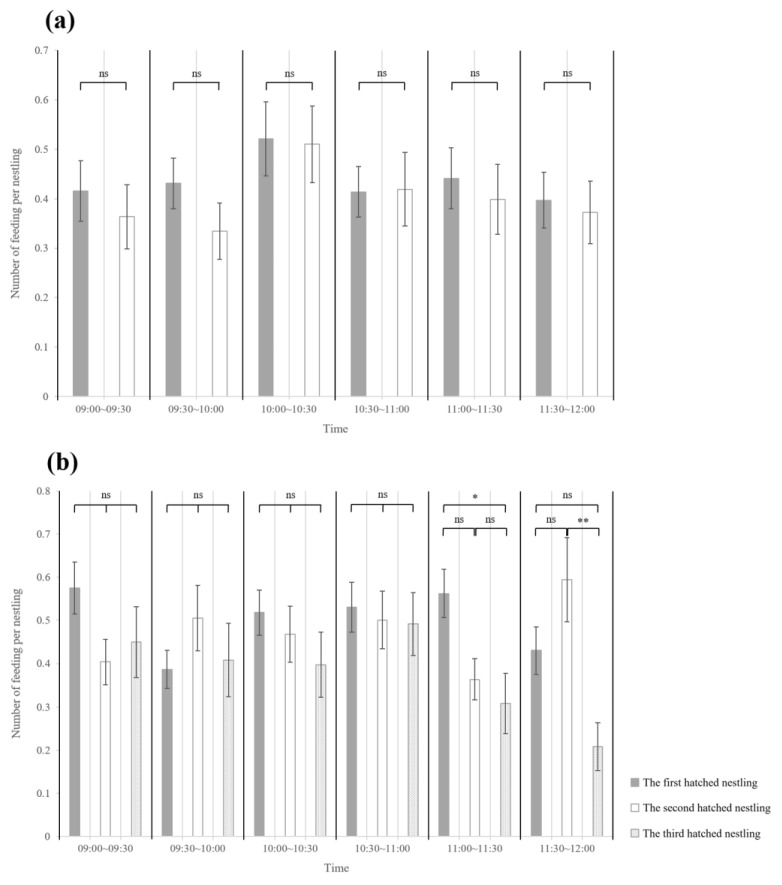
The average number of feedings per nestling during the period from 9:00 a.m. to 12:00 a.m., according to hatching order and time every 30 min, beginning at 9 a.m. (**a**) Nests that hatched over two days, (**b**) nests that hatched over three days. The black bar represents the first hatched nestling, the white bar represents the second hatched nestling, and the gray bar represents the third hatched nestling. (ns > 0.05, * *p* < 0.05, ** *p* < 0.01).

There was no statistically significant mean difference at all times in nests that hatched over two days (Figure 4a). In nests that hatched over three days, a significant difference between hatching order was observed only from 11:00 to 11:30 and 11:30 to 12:00 (Figure 4b). From 11:00 to 11:30, the average for the first hatched nestling was 0.56 ± 0.44 and the average for the third hatched nestling was 0.31 ± 0.55; a higher average number of feedings per nestling was observed for the first hatched nestling compared with the third hatched nestling. From 11:30 to 12:00, the average for the second hatched nestling was 0.59 ± 0.76 and the average for the third hatched nestling was 0.21 ± 0.43; a higher average number of feedings per nestling was observed for the second hatched nestling compared with the third hatched nestling.

## 4. Discussion

There are a few papers addressing brood reduction in passerines birds [34,35]. However, these studies focused on nest failure under asynchronous hatching conditions, not the feeding mechanism such as food distribution. In our study of the great tit, older nestlings were not targeted for selective distribution of food. As reported in a simulation study by Lee et al., 2020 [15], according to the brood reduction hypothesis, the survival rate of nestlings does not increase unless selective feeding of older nestlings is practiced in an environment of food scarcity. Therefore, the findings of this study support the concept that hypotheses, such as the brood reduction hypothesis, that propose food supply differences for nestlings do not adequately explain asynchronous hatching of passerines.

The results demonstrate that the difference in the average number of feedings per nestling according to the hatching order decreases with the passage of time from the day after all nestlings have hatched. A difference in the average number of feedings per nestling was observed between older nestlings and younger nestlings; we can presume that the reason for this is that the demand for food shows a gradual increase with increasing age of nestlings rather than selective distribution of food [36,37,38].

Examination of the food distribution for each hatching order over time at intervals of 30 min, beginning 9 a.m. when food distribution begins, showed that in terms of time, food was not selectively distributed to older nestlings. Therefore, even with regard to time, we can assume that interpretation of the asynchronous hatching of passerines according to the brood reduction hypothesis is problematic. From 11:00~11:30 and 11:30~12:00, based on our presumption, the statistically significant lower intake of the last hatched nestlings in nests that hatched over three days can be attributed to their younger age and smaller food requirements rather than lack of selective feeding [36,37,38].

Regarding the beak-open order factor, a type of begging, a higher average number of feedings per nestling was observed in nestlings who were the first to open their beak. Similar to the findings of this study, a study conducted by Moreno-Rueda et al. 2009 [39], Lee et al. 2012 [40], and Smith and Montgomerie 1991 [41] comparing the feeding rates of each nestling reported that the nestling that opened its beak first for begging received more food. In this study, a high average number of feedings per nestling was observed for the nestling that opened its beak first—that is, the first begging nestling; however, the results showed the even distribution of food to all nestlings regardless of age. According to our interpretation, the nestling’s begging indicated a reliable signal regarding its condition, and parents showed a rapid response [42,43,44]

The results of this study, which showed no significant correlation between nestling location and the average number of feedings per nestling, conflicted with the results reported by Moreno-Rueda et al. 2009 [39], who observed that nestlings that were closer in location to their parents received more food. Based on our assumption, these conflicting findings are a result of differences between studies and the method used for recording the location of nestlings. In this study, the distance between the hole where parents entered the nest box and the location of the nestling in the nest was recorded; however, in studies [39,45,46,47,48,49,50], the distance between the location where the parent landed for the purpose of feeding its nestling and the location of the nestling in the nest was recorded. Therefore, based on our assumption, the location where the parents actually landed to feed the nestling might be a more significant factor influencing the distribution of food than the location where the parents entered the nest. In addition, differences in the types of factors used in this study and those used in other studies might also explain this difference.

## 5. Conclusions

The results of this study, which showed that great tit parents did not practice selective feeding of older nestlings, indicate that using the brood reduction hypothesis to explain the asynchronous hatching of great tits is not reasonable. The following hypotheses do not propose strategies that focus on differences in supply of food to nestlings, such as the brood reduction hypothesis: according to the nest failure hypothesis, the breeding season is curtailed in order to minimize the risk of nestling predation [4,51]; the insurance hypothesis explains preparation for failure of hatching of the first laid egg [52]; according to the egg viability hypothesis, this is due to the decreasing survival rate of eggs over time [53,54,55]; according to the limited breeding opportunities hypothesis, locations for selection of a nest are limited and, therefore, rapid breeding is required when a location is identified [56]. To confirm the validity of these other hypotheses, it will be necessary to conduct further research to explain the asynchronous hatching in passerine species. If studies of good and poor years for food availability and sex-related differences between males and females are conducted in the future, it will be helpful to understand the brood reduction hypothesis and asynchronous hatching [57,58,59,60]. In addition, through manipulation of nestling position, it will be possible to clarify the feeding frequency according to our results and nestling position, which was different from previous studies.

## Figures and Tables

**Table 1:** In nests that hatched over two days and nests that hatched over three days, the average number of nestlings according to hatching order, the average clutch size, the average number of feedings per nestling during the period from 09:00 a.m. to 12:00 a.m., the average number of feedings per nestling from day 1 to day 6, the day after hatching of all nestlings.

Hatched Nest Types	Number of First Hatched Nestlings (Mean ± SE)	Number of Second Hatched Nestlings (Mean ± SE)	Number of Third Hatched Nestlings (Mean ± SE)	Clutch Size (Mean ± SE)	Number of Feedings per Nestling 09:00~12:00 a.m. (Mean ± SE)	Number of Feedings per Nestling Day 1~6 (Mean ± SE)
Nests that hatched over two days	4.75 ± 1.98	3.13 ± 1.97	-	7.88 ± 0.60	20.54 ± 11.56	123.25 ± 47.07
Nests that hatched over three days	3.80 ± 1.83	3.00 ± 1.67	1.20 ± 0.40	8.00 ± 1.26	23.05 ± 9.39	138.30 ± 30.11

**Table 2:** Correlation between hatching order, beak-open order, nestling age, nestling location, date variables, and the average number of feedings per nestling in nests that hatched over two days.

Linear	Resource	Unstandardized Coefficients		Standardized Coefficients	*t*	*p*	*F*	*R^2^*
		B	*SE*	*β*				
	Constant term	−4.023	2.986		−1.347	0.178		
Independent variable	hatching order	0.040	0.043	0.033	0.917	0.360	57.931	0.337
	beak-open order	0.650	0.042	0.533	15.638	<0.001		
	nestling age	0.077	0.012	0.225	6.319	<0.001		
	nestling location	−0.041	0.025	−0.056	−1.628	0.104		
	date	8.642 × 10^−5^	0.000	0.044	1.284	0.200		

**Table 3:** Correlation between hatching order, beak-open order, nestling age, nestling location, date variables, and the average number of feedings per nestling in nests that hatched over three days.

Linear	Resource	Unstandardized Coefficients		Standardized Coefficients	*t*	*p*	*F*	*R* ^2^
		B	*SE*	*β*				
	Constant term	5.104	3.624		1.408	0.159		
Independent variable	hatching order	−0.013	0.025	−0.017	−0.543	0.587	77.492	0.295
	beak-open order	0.696	0.037	0.525	18.971	<0.001		
	nestling age	0.045	0.011	0.128	4.210	<0.001		
	nestling location	0.041	0.022	0.051	1.849	0.065		
	date	0.000	0.000	−0.040	−1.448	0.148		

## Data Availability

The data presented in this study are available in Supplementary Material Table S1.

## Supplementary Materials

The following supporting information can be downloaded at: https://www.mdpi.com/article/10.3390/ani13091443/s1, Table S1: Original Data.
